# Pro-inflammatory interleukin-6 signaling links cognitive impairments and peripheral metabolic alterations in Alzheimer’s disease

**DOI:** 10.1038/s41398-021-01349-z

**Published:** 2021-04-28

**Authors:** Natalia M. Lyra e Silva, Rafaella A. Gonçalves, Tharick A. Pascoal, Ricardo A. S. Lima-Filho, Elisa de Paula França Resende, Erica L. M. Vieira, Antonio L. Teixeira, Leonardo C. de Souza, Julyanna A. Peny, Juliana T. S. Fortuna, Isadora C. Furigo, Debora Hashiguchi, Vivian S. Miya-Coreixas, Julia R. Clarke, Jose F. Abisambra, Beatriz M. Longo, Jose Donato, Paul E. Fraser, Pedro Rosa-Neto, Paulo Caramelli, Sergio T. Ferreira, Fernanda G. De Felice

**Affiliations:** 1 Institute of Medical Biochemistry Leopoldo de Meis, Federal University of Rio de Janeiro, Rio de Janeiro, RJ Brazil; 2 Centre for Neuroscience Studies, Queen’s University, Kingston, ON Canada; 3 Tanz Centre for Research in Neurodegenerative Diseases, University of Toronto, Toronto, ON Canada; 4 Translational Neuroimaging Laboratory, McGill Centre for Studies in Aging, Douglas Mental Health University Institute, Montreal, Quebec Canada; 5 Behavioral and Cognitive Neurology Research Group, Faculdade de Medicina, Universidade Federal de Minas Gerais, Belo Horizonte, MG Brazil; 6 Hospital das Clínicas, Universidade Federal de Minas Gerais, Belo Horizonte, MG Brazil; 7 Centre of Addiction and Mental Health (CAMH), Toronto, ON Canada; 8 Neuropsychiatry Program, Department of Psychiatry and Behavioral Sciences, McGovern Medical School, The University of Texas Health Science Center at Houston, Houston, TX USA; 9 Santa Casa BH Ensino e Pesquisa, Belo Horizonte, MG Brazil; 10 Institute of Biophysics Carlos Chagas Filho, Federal University of Rio de Janeiro, Rio de Janeiro, RJ Brazil; 11 Department of Physiology and Biophysics, Institute of Biomedical Sciences, University of São Paulo, São Paulo, SP Brazil; 12 Department of Physiology, Federal University of São Paulo, São Paulo, SP Brazil; 13 School of Pharmacy, Federal University of Rio de Janeiro, Rio de Janeiro, RJ Brazil; 14 Department of Neuroscience, Center for Translational Research in Neurodegenerative Disease University of Florida, Gainesville, FL USA; 15 Department of Medical Biophysics, University of Toronto, Toronto, ON Canada; 16 Department of Psychiatry, Queen’s University, Kingston, ON Canada; 17 Department of Biomedical and Molecuar Sciences, Queen’s University, Kingston, ON Canada

**Keywords:** Diseases, Molecular neuroscience

## Abstract

Alzheimer’s disease (AD) is associated with memory impairment and altered peripheral metabolism. Mounting evidence indicates that abnormal signaling in a brain-periphery metabolic axis plays a role in AD pathophysiology. The activation of pro-inflammatory pathways in the brain, including the interleukin-6 (IL-6) pathway, comprises a potential point of convergence between memory dysfunction and metabolic alterations in AD that remains to be better explored. Using T2-weighted magnetic resonance imaging (MRI), we observed signs of probable inflammation in the hypothalamus and in the hippocampus of AD patients when compared to cognitively healthy control subjects. Pathological examination of *post-mortem* AD hypothalamus revealed the presence of hyperphosphorylated tau and tangle-like structures, as well as parenchymal and vascular amyloid deposits surrounded by astrocytes. T2 hyperintensities on MRI positively correlated with plasma IL-6, and both correlated inversely with cognitive performance and hypothalamic/hippocampal volumes in AD patients. Increased IL-6 and suppressor of cytokine signaling 3 (SOCS3) were observed in *post-mortem* AD brains. Moreover, activation of the IL-6 pathway was observed in the hypothalamus and hippocampus of AD mice. Neutralization of IL-6 and inhibition of the signal transducer and activator of transcription 3 (STAT3) signaling in the brains of AD mouse models alleviated memory impairment and peripheral glucose intolerance, and normalized plasma IL-6 levels. Collectively, these results point to IL-6 as a link between cognitive impairment and peripheral metabolic alterations in AD. Targeting pro-inflammatory IL-6 signaling may be a strategy to alleviate memory impairment and metabolic alterations in the disease.

## Introduction

Alzheimer’s disease (AD) is a progressive neurodegenerative disorder with the highest prevalence in older adults^1^. Although AD is classically recognized as a disease that affects cognition, metabolic alterations have been also described in patients and in animal models^2–5^. Growing evidence indicates that miscommunication between the brain and periphery may be critical in the pathophysiology of AD^6,7^. One possibility is that damage to the blood-brain barrier (BBB)^6,7^ triggered by mediators and other signaling molecules released from peripheral tissues leads to brain inflammation in AD. Moreover, in AD brains, reactive astrocytes are associated with the chronic release of a variety of pro-inflammatory and toxic mediators^8,9^ that can elicit synaptic dysfunction^10–12^, alterations in the hypothalamus-pituitary-adrenal (HPA) axis^13^, and BBB dysregulation^14^. The chronic combination of central and peripheral inflammation may thus promote cognitive decline, brain and peripheral metabolic alterations in AD^2,10,11,15^.

The inflammatory mediators interleukin-6 (IL-6) and tumor necrosis factor-α (TNF-α) have been implicated in AD^10,11,16–18^. Our group has previously demonstrated that TNF-α mediates memory impairment and peripheral glucose intolerance by disrupting insulin signaling and activating cellular stress pathways in AD mouse models^2,11^. IL-6 is a component of early-stage amyloid plaque formation in AD brains^19^ and has been implicated in tau phosphorylation^20^, synapse loss, and learning deficits in mice^20–22^. Despite some controversy in the literature, previous meta-analyses have concluded that IL-6 is increased in both cerebrospinal fluid (CSF) and plasma of mild cognitive impairment (MCI) and AD patients compared to control individuals^23,24^. Although this suggests an association between IL-6 and AD pathogenesis, a causal link between IL-6, cognitive deficits, and peripheral metabolic dysregulation in AD remains to be established.

Using T2-weighted magnetic resonance imaging (MRI), we observed signs of probable inflammation (revealed by T2 hyperintensity signals) in the hypothalamus and hippocampus of AD patients compared to cognitively healthy controls. Hypothalamic and hippocampal T2 hyperintensities showed a positive correlation with IL-6 concentrations in the plasma, and a negative correlation with Mini-Mental State Examination (MMSE) scores and hypothalamic/hippocampal volumes. We observed the presence of histopathological AD hallmarks in *post-mortem* AD hypothalamus, including hyperphosphorylated tau, amyloid-β (Aβ) aggregates, and the presence of glial fibrillary acidic protein (GFAP) immunoreactivity surrounding parenchymal and vascular amyloid deposits. Moreover, IL-6-positive astrocytes and plaques were detected in AD hypothalamus and cortex, respectively, and increased IL-6 levels and signaling were found in AD cortex, as well as in the hippocampus and hypothalamus of animal models of AD. Increased plasma IL-6 correlated inversely with cognitive performance and hypothalamic/hippocampal volumes in a cohort of AD patients and cognitively healthy controls, and the neutralization of IL-6 in the brains of AD mouse models rescued memory deficits, peripheral glucose intolerance and circulating IL-6 levels. Our findings establish dysregulated IL-6 signaling as a key mechanism linking memory/cognitive impairment and metabolic dysregulation in AD.

## Methods

### Subjects

Patients with dementia due to AD were recruited at a public university-based memory clinic (Hospital das Clínicas, Federal University of Minas Gerais, Belo Horizonte, Brazil), and were diagnosed following the National Institute of Aging and Alzheimer’s Association criteria^25^. Cognitively healthy controls were recruited from the local community. Individuals were matched to patients by age, sex, education, and socioeconomic status (Supplementary Table 1). Exclusion criteria for both patient and control groups included the presence of other diagnosed neurological syndromes. Cognition was evaluated through the Mini-Mental State Examination (MMSE)^26^, the verbal phonemic fluency^27^, the Brief Cognitive Battery^28^, and the Frontal Assessment Battery (FAB)^29^. These procedures, as well as the MRI and all laboratory tests (see below), received approval from the institution’s ethics committee. Prior to joining the study, participants signed written informed consent.

### Human brain samples

Paraffin-embedded *post-mortem* samples of the lateral hypothalamus and the cingulate cortex from AD and controls were obtained from the Maritime Brain Tissue Bank (Nova Scotia, CA). Frozen brain tissue from AD and controls (Supplementary Table 2 for demographics) were acquired from the University of Kentucky Alzheimer’s Disease Center (UKADC) Tissue Bank (Lexington, USA). Sample collection and experimental procedures followed protocols approved by Institutional Review Board.

### Magnetic resonance imaging

Subjects underwent brain MRI scans in a 3 Tesla Philips Achieva scanner. Three-dimensional T1-weighted images were acquired in the sagittal plane with spin-echo echoplanar sequences, TR/TE = 16/4 ms, matrix = 240 × 240, slice thickness=1.0 mm, 1.0 mm gap between slices and flip angle of 8°. T2 images were obtained in axial plane with single-shot, spin-echo echoplanar sequences (TR/TE = 140,000/120 ms, Echo train length=27 mm, FO = 240 mm, matrix=352 × 212 (reconstructed 512 × 512), slice thickness = 5.0 mm, 6.0 mm gap between slices). Regions-of-interest (ROIs) were tailored from validated brain Atlas^30,31^. Briefly, T1-weighted MRIs were corrected for field distortions, non-uniformity corrected, segmented, and non-linearly transformed to the MNI reference space using the CIVET pipeline, according to our standard protocol^32^. Then, we transformed the Brain Atlas ROIs from the MNI reference space to each individual native space using the aforementioned non-linear transformation. Transformed ROIs were visually inspected by two neurologists to confirm anatomical accuracy. T2 image signal intensity, previously found to be a proxy of brain gliosis^33–37^, was measured in the hypothalamus, hippocampus, and putamen ROIs. T2 signal intensity ratios were then calculated by comparing mean signal intensities in hypothalamus or hippocampus ROIs with the mean signal intensity in the control putamen ROI, as previously described^38^.

### Immunofluorescence

Five µm sections of paraffin-embedded tissue underwent epitope retrieval with boiling citrate buffer for 20 min followed by blockage with 5% normal goat serum (NGS) and 5% bovine serum albumin (BSA) diluted in 0.05% Triton X-100 Phosphate-buffered saline (PBS). Primary antibodies against Aβ, 6E10 (1:200; Biolegend, San Diego, CA; Cat#803014) and ab134022 (1:200; Abcam, Cambridge, UK), phospho-TauSer202Thr205, AT8 (1:200; Invitrogen, Waltham, MA; Cat#MN1020) and GFAP (1:500; Cell signaling, Danvers, MA) were diluted in blocking buffer. Brain sections were incubated with primary antibody for 16 h at 4 °C. For IL-6/GFAP double immunofluorescence, primary antibodies against IL-6 (1:1,000; Abcam; ab6672) and GFAP (1:1,000; Cell signaling; Cat#3670) were incubated at 4 °C for 3 consecutive days. Sections were incubated with the secondary antibodies Cy3- (1:500; Abcam), Cy5- (1:500; Abcam), or AlexaFluor 488-conjugated (1:1,000; Thermo Fisher Scientific, Waltham, MA; Cat#A11034) for 2 h at room temperature. Prolong Gold Antifade with DAPI (Invitrogen) was used to mount the slides. The Zeiss Axio Observer Z1 microscope was used for imaging. The absence of nonspecific labeling was confirmed by not including the primary antibodies in the reaction (data not shown).

### Animals

Institutional Animal Care and Use Committee of the Federal University of Rio de Janeiro approved all experiments with animals included in this study. Three-month-old C57BL/6 male mice were obtained from our own facility and were randomly assigned to receive either intracerebroventricular (i.c.v.) infusions of Aβ oligomers (AβOs) or vehicle (Veh) (see below). APP/PS1 transgenic mice (APPswe/PS1ΔE9; RRID: MMRRC_034829-JAX) were maintained in a hemizygote state by crossing to C57BL/6 mice. Eleven‐month‐old APP/PS1 and wild‐type (WT) littermate male mice were used in this study. All animals were housed in groups of five per cage in a room under a 12‐h light/dark cycle, and with free access to food and water. Researchers were blinded to the experimental conditions. Animals were deeply anesthetized with xylazine (10 mg/kg) and ketamine (100 mg/kg) prior to termination by decapitation or transcardial perfusion with PBS and 4% paraformaldehyde solution in PBS.

### Enzyme-linked immunosorbent assays (ELISAs)

Human *post-mortem* tissues were homogenized in Tris-buffered saline (TBS) with phosphatase and protease inhibitors. IL-6 was detected using the DuoSet Human IL-6 ELISA kit (DY206-05; R&D Systems, Minneapolis, MN). For measurements in mice, blood was collected either via cardiac puncture using heparin-containing syringes (for IL-6 determination) or from the tail vein using EDTA-coated microvettes (Sarstedt, Numbrecht, Germany) (for insulin/leptin determination) after 4 h of fasting. ELISAs were performed using plasma and according to manufacturers’ instructions: DuoSet mouse IL-6 ELISA kit (DY406-05; R&D Systems), ultrasensitive insulin ELISA (ALPCO Diagnostics, Salem, NH), and mouse leptin ELISA (Crystal Chem Inc, Elk Grove Village, IL).

### Cytometric beads array

IL-6 in human plasma was analyzed using the cytometric bead array (CBA) kit according to instructions provided by the manufacturer (Becton & Dickinson, Franklin Lakes, NJ). A FACS CANTO II flow cytometer was used to acquire data, that were subsequently analyzed using the FCAP Array software (Becton & Dickinson).

### Intracerebroventricular infusion of AβOs

Synthetic Aβ_1–42_ peptide (American Peptide) was used to prepare the AβOs as originally described^39^, and each preparation was characterized by size-exclusion gel filtration^40^. AβOs were stored at 4 °C and were used within 48 h of preparation. 100 pmol of AβOs in a total volume of 1.5 μl, or an equal volume of vehicle, was i.c.v. infused unilaterally once into C57BL/6 mice, as previously described^40^.

### Intracerebroventricular treatments with anti-IL-6 or AG490

APP/PS1 or WT mice were randomized between experimental group conditions and received four unilateral i.c.v. infusions of 300 ng anti-IL-6 (R&D Systems) or an irrelevant antibody (anti-GFP, Invitrogen) every other day, in a total volume of 1.5 μl. Novel object recognition (NOR) and glucose tolerance (GTT) tests (see below) were performed 24 h after the third and fourth infusions of anti-IL-6 or anti-GFP, respectively. A total of 5 nmol of AG490 (Millipore, Burlington, MA) in a total volume of 1 μl was i.c.v. infused unilaterally into C57BL/6 mice thirty minutes after AβOs or Veh were i.c.v. infused. GTT and NOR were performed 36 and 48 h after treatments, respectively.

### Primary hippocampal cultures and immunocytochemistry

18 day-old Wistar rat embryos had their hippocampi dissected and cultures were prepared as described in ref. ^41^. Cells were maintained in culture for 18 days and were subsequently incubated with 500 nM of AβOs, or a corresponding volume of vehicle for 24 h at 37 °C. Immunocytochemistry was performed as previously described^42^ using MAP2 (1:200; Santa Cruz Biotechnology; Cat#sc20172) and pSTAT3 antibodies (1:500; Santa Cruz Biotechnology; Cat#sc8059) at 4 °C for 16 h, and Alexa-conjugated secondary antibodies at room temperature for 2 h. The Zeiss Axio Observer Z1 Microscope with an Apotome module was used to image the cells.

### Western blots

Hippocampi and hypothalami were dissected, frozen in dry ice, and homogenized as described^43^. Protein content was separated on SDS-PAGE gels (4–20%) and transferred to a nitrocellulose membrane. Membranes were incubated for 16 h at 4 °C with pSTAT3 (1:500; Cell Signaling; Cat#9131 S), SOCS3 (1:500; Cell Signaling; Cat#2923) or β‐actin (1:10,000; Cell Signaling; Cat#12262) antibodies in blocking buffer with 3% bovine serum albumin (BSA). Subsequently, membranes were incubated with horseradish peroxidase‐conjugated secondary antibody (1:30,000–50,000; Thermo Fisher Scientific), or with IRDye800CW‐ or IRDye680RD‐conjugated secondary antibodies (1:10,000; Licor) in blocking buffer at room temperature for 2 h. ECL reagent or SuperSignal West Femto (Thermo Fisher Scientific) were used to develop chemiluminescence. Signals were quantified on ChemiDoc (BioRad, Hercules, CA) or Odyssey CLx apparatuses (Licor, Lincoln, NE).

### In situ hybridization

Brains were processed as described^44^. SOCS3-^35^S-labeled riboprobes were generated from cDNA templates corresponding to positions 634-981 from the coding sequence of the mouse *Socs3* gene (GenBank accession number NM_007707.3). Hybridization buffer was used to dilute cRNA probes. Sections were hybridized overnight at 56 °C and mounted onto SuperFrost Plus slides (Fisher Scientific). Slices underwent a dehydration step that involved increasing concentrations of ethanol and clearing in xylene for 5 min. The sections were dipped into NTB2 photographic emulsion (Kodak), dried, and stored at 4 °C for approximately 30 days in slide boxes protected from light and containing a desiccant. D-19 developer (Kodak) was used to further develop the slices. After the second step of dehydration, as described above, slices were coverslipped with DPX (Sigma-Aldrich). The Axio Imager A1 (Zeiss) microscope was used to image the slides.

### RNA extraction and quantitative PCR (qPCR)

Tissues were dissected and submerged in RNAlater solution (Thermo Fisher Scientific). Samples were homogenized as described^43^. Two μg of RNA were used to synthesize the cDNA using the SuperStrand III Reverse Transcriptase kit (Invitrogen). The Applied Biosystems 7500 Real-Time PCR system in combination with the Power SYBR kit (Applied Biosystems) were used to analyse the expression of genes of interest. β-actin or β-tubulin were used as endogenous controls. Supplementary Table 3 provides the primer pair sequences used. Fold changes in gene expression were calculated using cycle threshold (*C*_*t*_) values and the 2^-ΔΔ*Ct*^ method^45^.

### Novel object recognition (NOR) task

NOR was carried out as previously described^11,40^. Training and test sessions were 5-min long. During the training, animals were placed in the center of an open field arena where they could freely explore two identical objects. The time spent exploring each object was measured by an experienced researcher. During the test session (one hour later), animals were reintroduced to the arena, where they could now explore one novel object and one of the two familiar objects previously used in the training session. An experienced researcher measured the time exploring familiar and novel objects. Results represent the percentage of time exploring each object in relation to the total exploration time of both objects. The percentage of time spent exploring the novel object was compared to the chance level of 50% using one-sample Student’s t-test.

### Glucose tolerance test (GTT)

Following 12 h of fasting, 1 g/kg body weight of glucose was intraperitoneally injected. Blood glucose levels were measured from the tail vein at different times thereafter using the One‐Touch^®^ Ultra^®^ glucometer (Johnson & Johnson).

### Statistics

Values are expressed as means ± standard error of the mean (SEM). Statistical analyses were performed using GraphPad Prism 6 (GraphPad Software) and significances were determined by Student’s t-test, one- or two-way ANOVA followed by Dunnet’s or Tukey’s post-hoc tests, as appropriate. Welch’s correction was used when variances differed between groups. Correlation analyses were determined using Pearson’s correlation coefficient or Spearman’s rank correlation coefficient were calculated when variables were not normally distributed. Sample sizes were estimated from pilot studies and previous experience. No algorithm or software was used for experimental group randomization.

## Results

### T2-weighted signal hyperintensities in the AD hypothalamus and hippocampus correlate inversely with MMSE scores and gray matter densities

**Fig. 1:**
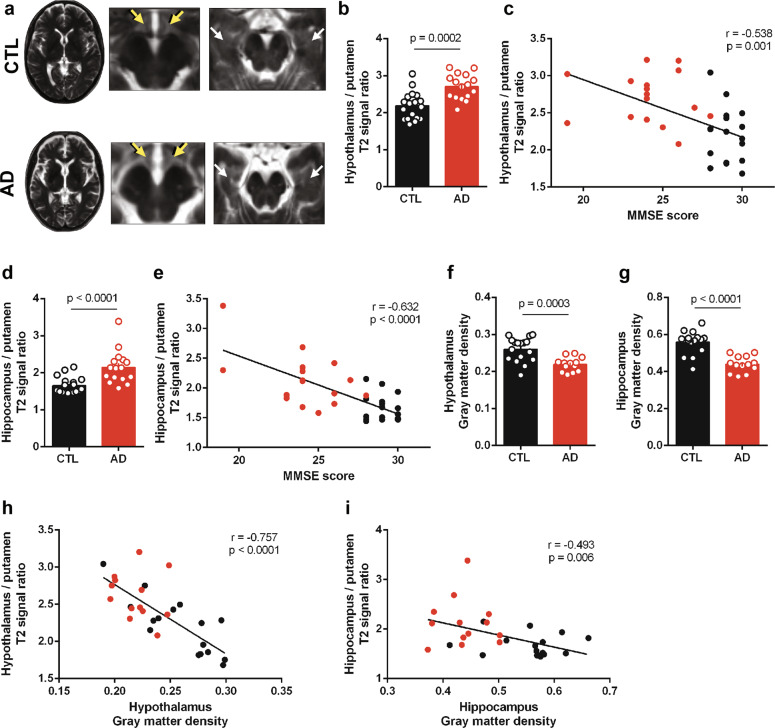
T2-weighted signal hyperintensities in the AD hypothalamus and hippocampus correlate inversely with MMSE scores and gray matter densities. **a** Representative transversal (left) and coronal (right) T2-weighted images from control (CTL, top) and AD (bottom) patients. Arrows indicate the placement of right and left ROIs in the hypothalamus (yellow arrows) and hippocampus (white arrows). **b** Bars represent means ± SEM of T2-intensity signal ratios (hypothalamus relative to putamen) in control subjects (*n* = 18) and AD patients (n = 16); symbols represent individual subjects; *p*-value was calculated from student’s t-test and is shown in the graph. **c** Correlation between hypothalamus/putamen T2-intensity signal ratios and MMSE scores. Pearson correlation coefficient (r) and *p*-value are shown in the graph (*n* = 34). **d** Bars represent means ± SEM of T2-intensity signal ratios (hippocampus relative to putamen) in control subjects (*n* = 18) and AD patients (*n* = 16); symbols represent individual patients; p-value (Mann-Whitney test) is shown in the graph. **e** Correlation between hippocampus/putamen T2-intensity signal ratios and MMSE scores. Spearman correlation coefficient (r) and p-value are shown in the graph (*n* = 34). Bars represent means ± SEM of hypothalamic (**f**) and hippocampal (**g**) gray matter densities measured by voxel-based morphometry in control subjects (*n* = 17) and AD patients (*n* = 13); symbols represent individual patients; p-value (Student’s t-test) is shown in graph. **h** Correlation between hypothalamus gray matter density and hypothalamus/putamen T2-intensity signal ratios. Pearson correlation coefficient (r) and *p*-value are shown in the graph (*n* = 30). **i** Correlation between hippocampus gray matter density and hippocampus/putamen T2-intensity signal ratios. Spearman correlation coefficient (r) and *p*-value are shown in the graph (*n* = 30).

We initially sought to investigate whether AD patients exhibited signs of probable hypothalamic inflammation. Previous findings from our group have shown elevated inflammatory markers in the hypothalamus of rodent and non-human primate models of AD^2^. We first focused on signs of hypothalamic inflammation by examining T2-weighted MRI signal intensities^35,38,46,47^ in images obtained from a cohort of 16 AD patients and 18 control individuals (Fig. 1a; Supplementary Table 1). We defined regions of interest in the hypothalamus and in the putamen (used as a control region) and compared the ratios of hypothalamus/putamen T2-weighed intensities in AD versus controls. We found a significant increase in hypothalamus/putamen ratios in AD compared to control subjects (Fig. 1b). We further observed that hypothalamic T2 hyperintensities showed an inverse correlation with MMSE scores in our study sample (Fig. 1c).

Because brain inflammation, particularly in brain areas involved in learning and memory, has been implicated in cognitive impairment and neuronal dysfunction in AD, we further examined hippocampal T2-weighted signals in our sample. We detected a significant increase in the hippocampus/putamen signal ratio in the AD group compared to healthy controls (Fig. 1d), and an inverse correlation between hippocampus/putamen T2 signal ratios and MMSE scores (Fig. 1e). Results further revealed a strong correlation between hypothalamic and hippocampal T2 signal hyperintensities (Supplementary Fig. 1). These results indicate that these signs of probable inflammation correlate with impaired cognitive function and occur in parallel in the AD hippocampus, a key memory center, and hypothalamus, a brain region centrally involved in peripheral metabolic control.

Further, we used voxel-based morphometry (VBM) to assess hypothalamic and hippocampal volumes in the same cohort. AD patients exhibited decreased gray matter densities in the hypothalamus (Fig. 1f) and hippocampus (Fig. 1g). These findings are in accordance with previous studies^48–50^. Additionally, we observed a negative correlation between volume and T2 signal hyperintensities in both brain regions (Fig. 1h, i).

### Intraneuronal hyperphosphorylated tau aggregates, Aβ deposits and inflammatory markers in *post-mortem* AD hypothalamus

**Fig. 2:**
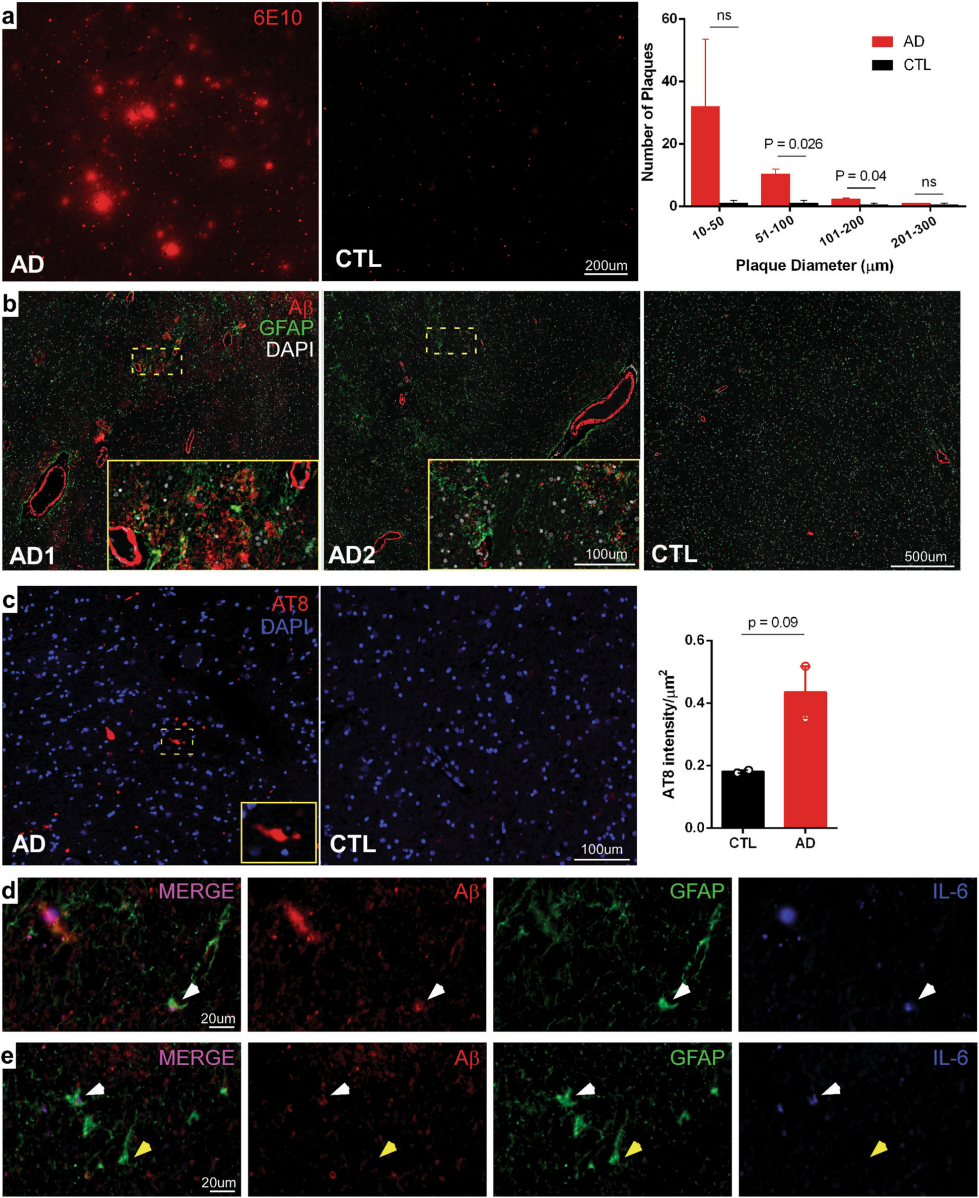
Intraneuronal hyperphosphorylated tau aggregates, Aβ deposits and inflammatory markers in *post-mortem* AD hypothalamus. **a** Representative photomicrographs showing immunostaining of Aβ deposits (6E10 antibody; red) in the lateral hypothalamus of AD cases (AD1: Female 77 years old; AD2: Female, 88 years old; AD3: Female, 82 years old) and age-matched control (CTL1: Female, 71 years old; CTL2: Female, 80 years old). Graph represents means ± SEM of number of plaques in AD patients or control individuals; p-value was calculated from student’s t-test, and is shown in the graph. **b** Representative photomicrographs of the lateral hypothalamus showing double immunofluorescence staining for Aβ (Abcam ab134022; red) and astrocytes (GFAP; green) in *post-mortem* AD (AD1: Female 77 years old; AD2: Female, 88 years old) and age-matched control (CTL: Female, 71 years old). Magnified images of specific fields are indicated by the dashed rectangles in the main panels. **c** Representative photomicrographs of the lateral hypothalamus showing positive intraneuronal immunostaining for AT8 (p-tau antibody; red) in neurons in AD cases (AD1: Female, 82 years old; AD2: Female 77 years old) and absence of AT8-positive cells in age-matched control (CTL1: Female, 71 years old; CTL2: Female, 80 years old). Graph represents means + SEM of number of plaques in AD patients or control individuals; p-value (Student’s t-test) is shown in the graph. **d**, **e** Representative micrographs of triple immunofluorescence staining for Aβ (Abcam ab134022; red), astrocytes (GFAP; green), and IL-6 (blue) in AD lateral hypothalamus (Female, 77 years old). White arrows indicate a cell with triple staining for GFAP, Aβ and IL-6, and yellow arrows show a GFAP-positive cell that does not colocalize with either Aβ or IL-6 immunoreactivities.

We then moved to investigate the presence of neuropathological hallmarks of AD in the lateral hypothalamus of *post-mortem* AD brain tissue. We detected the presence of dense Aβ aggregates and strong vascular deposition of Aβ in AD hypothalamus (Fig. 2a, b). Immunohistochemistry using the AT8 antibody revealed the presence of intracellular aggregates of hyperphosphorylated tau in hypothalamic neurons and an increasing trend of AT8 fluorescence intensity in AD (Fig. 2c).

GFAP-positive astrocytes were found surrounding parenchymal Aβ plaques and vascular Aβ deposits in AD hypothalamus (Fig. 2b). Since astrocytes have been implicated in brain inflammation and have been shown to be the primary producers of IL-6 in AD^9,51^, we investigated if IL-6 co-localizes with astrocytes surrounding Aβ deposits in the AD hypothalamus. Indeed, IL-6 immunoreactivity was detected in hypothalamic GFAP-positive cells that were also positive for Aβ in the AD brain (Fig. 2d, e).

### IL-6 is upregulated in AD brain and plasma, and correlates positively with brain inflammation and inversely with MMSE scores

**Fig. 3:**
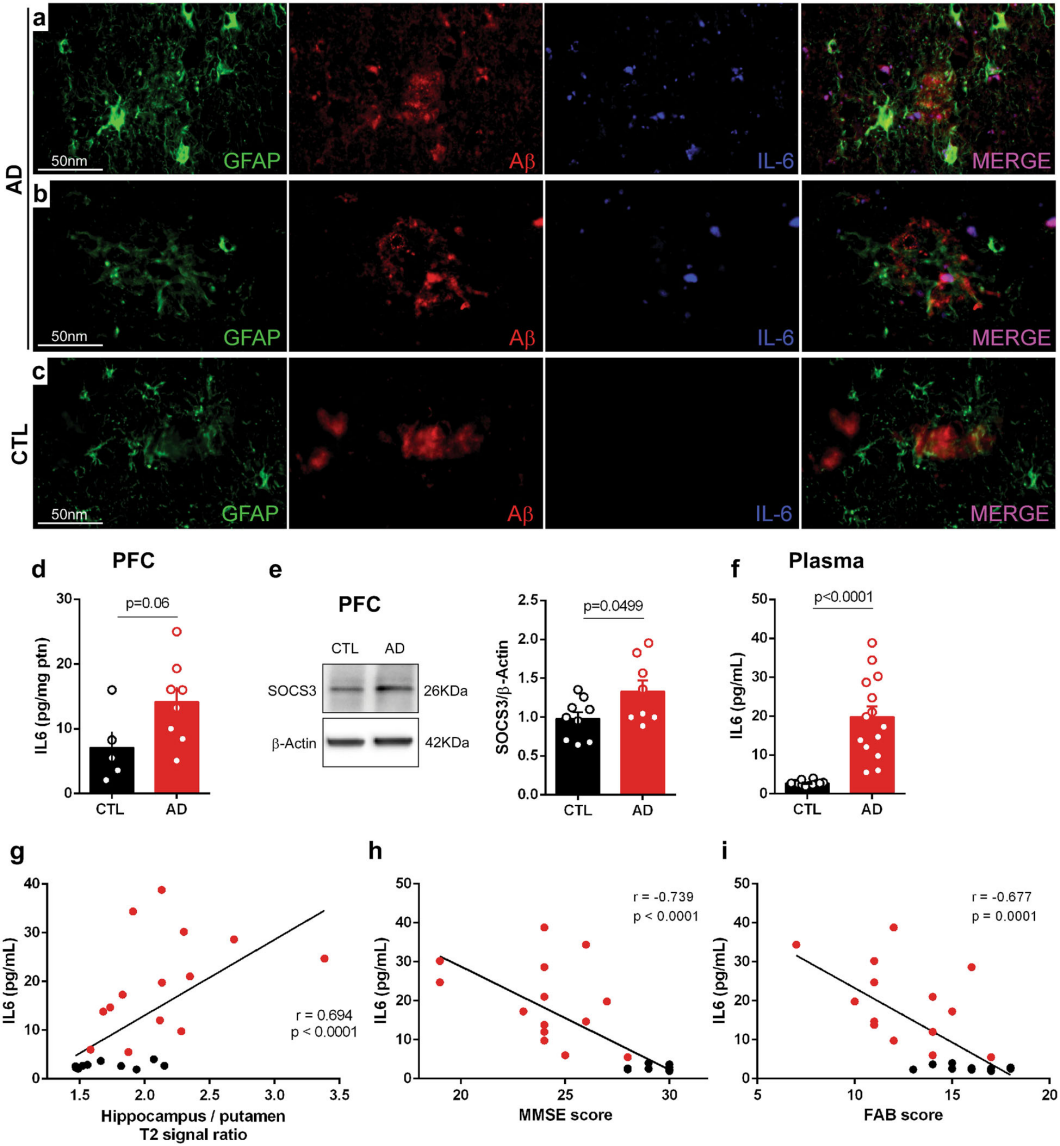
IL-6 is upregulated in AD brain and plasma, and correlates inversely with MMSE. Representative photomicrographs of triple immunofluorescence staining for Aβ (Abcam ab134022; red), astrocytes (GFAP; green), and IL-6 (blue) in *post-mortem* cingulate cortex from AD subjects (AD1: Female, 82 years old; AD2: Female, 88 years old) (**a**, **b**) and an age-matched control (CTL: Female, 80 years old) (**c**). **d** Means ± SEM of IL-6 protein levels measured by ELISA in *post-mortem* prefrontal cortex from AD (*n* = 8) or control subjects (*n* = 5); symbols represent different individuals; *p*-value was calculated from student’s t-test and is shown in the graph. **e** Representative immunoblot of SOCS3 (β-actin used as loading control) in *post-mortem* prefrontal cortex (PFC) from AD (*n* = 8) or control (*n* = 9) subjects. Graph shows means ± SEM of SOCS3/β-actin ratios; symbols represent different individuals; *p*-value (Student’s t-test) is shown in the graph. **f** Means ± SEM of plasma IL-6 in AD patients (*n* = 12) or control individuals (*n* = 14) from the same cohort that was studied by MRI in Fig. 1; symbols represent different individuals; p-value was calculated from unpaired t-test with Welch’s correction, and is shown in the graph. **g** Correlation between plasma IL-6 and hippocampus/putamen T2-intensity signal ratios for patients in the studied cohort (*n* = 26). Spearman correlation coefficient (r) and *p*-value are shown in the graph. **h**, **i** Correlations between plasma IL-6 and MMSE or FAB scores for patients in the studied cohort. Symbols represent different individuals (*n* = 26). Pearson correlation coefficients (r) and *p*-values are shown in the graphs.

We further examined the cingulate cortex, a brain region prominently affected in AD^52,53^, in the same *post-mortem* brains described above. The analysis revealed IL-6 immunoreactivity in Aβ plaques of AD brains, but not in plaques present in age-matched control brains (Fig. 3a–c).

To determine whether IL-6 was upregulated in AD brains, we measured IL-6 levels in pre-frontal cortex (PFC) homogenates from AD and control brains (Supplementary Table 2; Fig. 3d). This showed an increase in IL-6 in the AD PFC compared to controls. We further detected an increase in suppressor of cytokine signaling 3 (SOCS3), a downstream mediator of IL-6 signaling, in the AD PFC relative to control (Fig. 3e).

A marked increase in IL-6 was further detected in the plasma of the AD patients (Fig. 3f) studied by MRI in Fig. 1. Plasma IL-6 correlated positively with hippocampal (Fig. 3g) T2-weighted signals and correlated inversely with performance in the MMSE (Fig. 3h), FAB (Fig. 3i), category fluency (Supplementary Fig. 2a) tests, as well as with hypothalamic and hippocampal gray matter densities (Supplementary Fig. 2b, c). These results suggest a correlation between brain/peripheral inflammation, neurodegeneration, and impaired cognitive performance.

### Brain IL-6 signaling is upregulated and mediates memory impairment in AD mouse models

**Fig. 4:**
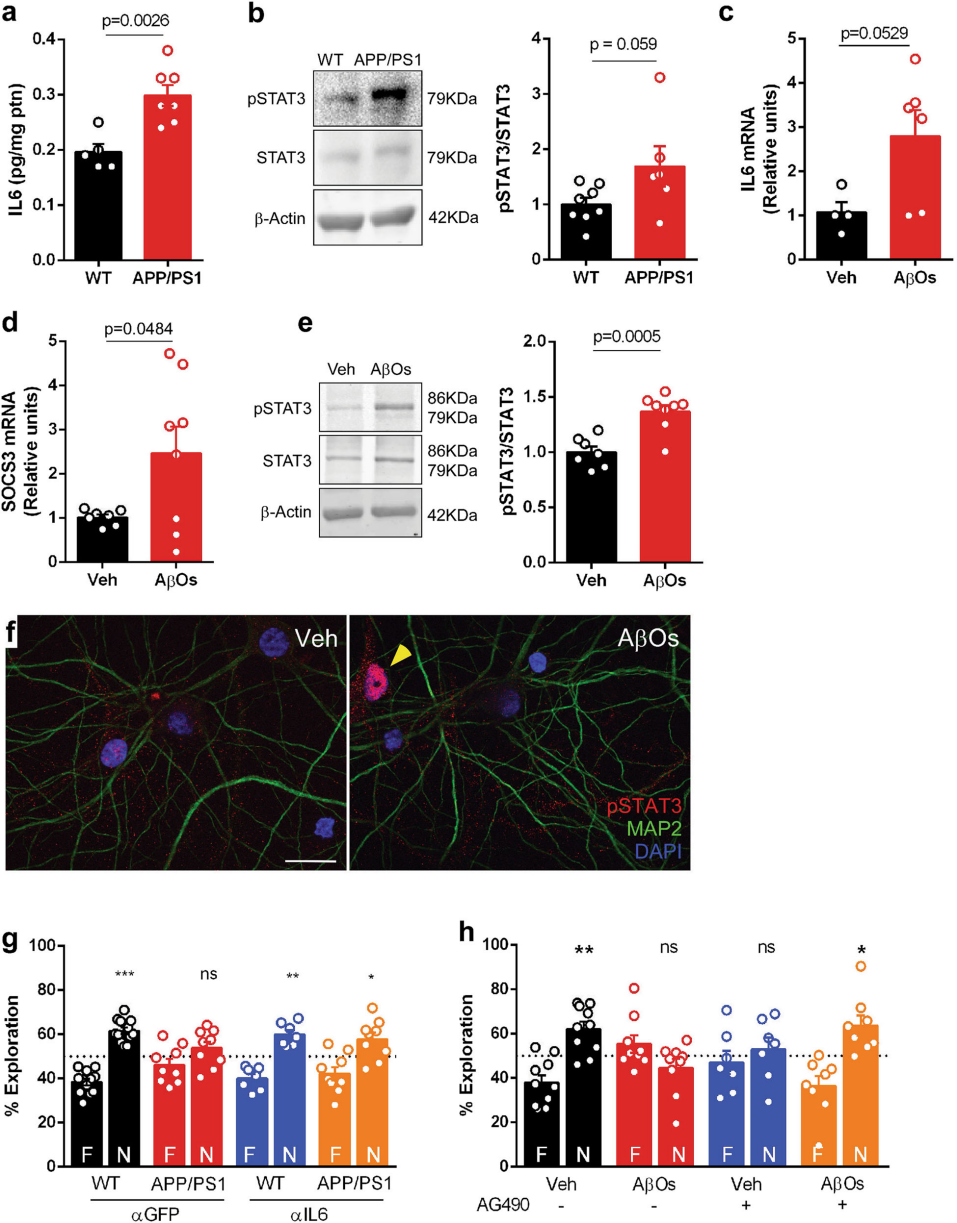
Brain IL-6 signaling is upregulated and mediates memory impairment in AD mouse models. **a** Means ± SEM of IL-6 protein levels in the hippocampus of 11-month-old APP/PS1 (*n* = 7) and WT littermates (*n* = 5); symbols represent individual animals; p-value (Student’s t-test) is shown in graph. **b** Representative immunoblot of pSTAT3, STAT3, and β-actin (used as a loading control) in the hippocampus of 11-month-old APP/PS1 (*n* = 6) and WT mice (*n* = 8). Graph shows pSTAT3/STAT3 ratio; symbols represent individual animals; *p*-value (Student’s t-test) is shown in graph. **c**, **d** Means ± SEM of IL-6 (*n* = 4 Veh; 6 AβOs) and SOCS3 expression (*n* = 7 Veh; 8 AβOs) (mRNA levels, expressed as fold-change relative to vehicle-infused mice) in mouse hippocampus 24 h after i.c.v. infusion of AβOs. β-actin was used as a housekeeping control. Symbols represent individual animals; *p*-values were calculated from student’s t-test and unpaired t-test with Welch’s correction, and are shown in graphs. **e** Representative immunoblot of pSTAT3, STAT3, and β-actin (loading control) in mouse hippocampus 24 h after i.c.v. infusion of AβOs (n = 8) (or vehicle, *n* = 7). Graph shows pSTAT3/STAT3 ratio; symbols represent individual animals; p-value (Student’s t-test) is shown in graph. **f** Representative immunofluorescence staining for pSTAT3 (red), MAP2 (green), and DAPI (blue) in a primary hippocampal culture exposed to AβOs (or vehicle) for 24 h. Yellow arrowhead indicates pSTAT3 staining (red) in the nucleus of a cell that is not positive for MAP2 (green). **g** Novel Object Recognition (NOR) task with 11-month-old male APP/PS1 mice and WT littermates (*n* = 7–12) after 3 i.c.v. injections of anti-IL-6 (αIL6) or anti-GFP (αGFP), as indicated. Symbols represent individual mice, *n* = 7–12 per experimental condition; *p < 0.05, **p < 0.01, ***p < 0.001, one-sample Student’s t-test. **h** NOR task with 3-month-old mice i.c.v-infused with AβOs (or vehicle) and AG490. Bars represent means ± SEM. Symbols represent individual mice, *n* = 7–10 per experimental condition; *p < 0.05, **p < 0.01, one-sample Student’s t-test. Data are representative of at least two independent experiments.

Next, we sought to evaluate brain IL-6 signaling and the involvement of this pathway in memory function in mouse models of AD. We first observed increased IL-6 and STAT3 activation in the hippocampi of APP/PS1 mice compared to WT animals (Fig. 4a, b).

To directly investigate the role of Aβ in hippocampal upregulation of IL-6 signaling, we carried out experiments using AβOs, toxins involved with AD pathogenesis^54,55^. We observed elevated expression of IL-6 and SOCS3 (Fig. 4c, d) and activation of STAT3 (pSTAT3; Fig. 4e) in the hippocampi of mice 24 h after a single intracerebroventricular (i.c.v.) infusion of AβOs. In primary hippocampal cultures exposed to AβOs, pSTAT3 was prominently located in the nuclei of non-neuronal cells exposed to AβOs (Fig. 4f), which is consistent with its action as a transcription factor.

To investigate the involvement of brain IL-6 signaling in memory impairment in AD mouse models, we first treated APP/PS1 mice via i.c.v. with an IL-6 neutralizing antibody^56^ and assessed memory using the NOR task. APP/PS1 mice treated with anti-IL-6 exhibited similar performance in the NOR task as WT mice, indicating reversal of memory deficits (Fig. 4g). As expected, APP/PS1 mice treated with a control irrelevant antibody (anti-GFP) failed to discriminate between new and familiar objects in NOR task (Fig. 4g).

To further examine the impact of increased central IL-6/STAT3 signaling on memory deficits, we treated AβO-infused mice via i.c.v. with the STAT3 inhibitor, AG490. Consistent with our previous reports^11,40^, AβO-infused mice showed impaired memory in the NOR task (Fig. 4h). AβO-induced memory impairment was prevented by treatment with AG490 (Fig. 4h). In line with a previous report^57^, AG490 alone caused a deficit in NOR memory in vehicle-infused mice (Fig. 4h). Control measurements showed no innate object preference or differences in distance, time spent at the periphery or center of the arena, velocity, and total time spent exploring objects between experimental groups, indicating no impact of treatments on locomotor or exploratory activities of mice (Supplementary Figs. 3 and 4).

### Central IL-6 is linked to peripheral metabolic dysfunction in AD mouse models

**Fig. 5:**
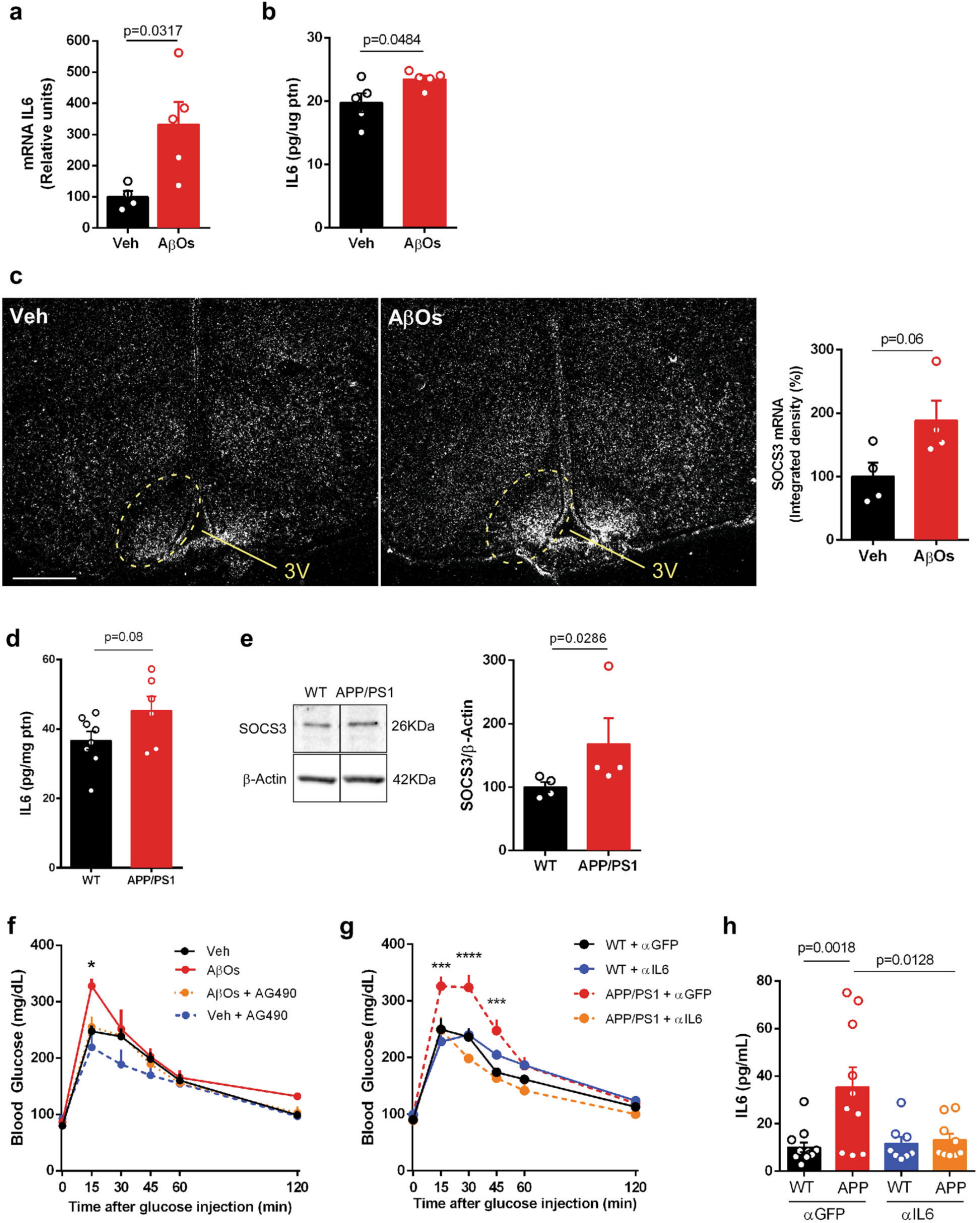
Central IL-6 promotes peripheral metabolic dysfunction in AD mouse models. **a** Expression of IL-6 in the mouse hypothalamus 4 h after i.c.v. infusion of AβOs (*n* = 5) (or vehicle, *n* = 4). β-actin was used as housekeeping control. Bars represent means ± SEM; symbols correspond to individual mice; *p*-value (Student’s t-test) is shown in graph. **b** IL-6 in the mouse hypothalamus 4 h after AβOs (*n* = 5) or vehicle (*n* = 5) were i.c.v.-infused. Bars represent means ± SEM; symbols correspond to individual mice; p-value (Student’s t-test) is shown in graph. **c** Representative in situ hybridization images for SOCS3 in the mouse hypothalamus 7 days after AβOs (*n* = 4) or vehicle (*n* = 4) were i.c.v.-infused. Graph shows integrated densities (means ± SEM) in the regions indicated by dashed ellipses in AβO- or vehicle-infused mice. Symbols correspond to individual mice; *p*-value (Student’s t-test) is shown in the graph. **d** IL-6 in hypothalamic homogenates from 11-month-old APP/PS1 mice (*n* = 6) or WT litttermates (*n* = 8). Bars correspond to means ± SEM. Symbols correspond to individual mice; *p*-value (Student’s t-test) is shown in the graph. **e** Representative immunoblot for SOCS3 in the hypothalamus of 11-month-old male APP/PS1 mice (*n* = 4) or WT littermates (*n* = 4). Bars in graph correspond to means ± SEM. Symbols correspond to individual mice; p-value (Mann–Whitney test) is shown in graph. **f** Glucose tolerance test in WT mice 36 h after i.c.v. infusion of AβOs (or vehicle) and treatment with AG490. Symbols correspond to individual mice and represent means ± SEM, *n* = 4–6 per experimental group; *p < 0.05 from two-way ANOVA followed by Tukey’s *post-hoc* test comparing Veh vs. AβOs and AβOs vs. AβOs + AG490). **g** Glucose tolerance test in 11-month-old APP/PS1 mice after 4 i.c.v. injections of anti-IL-6 (αIL6) or anti-GFP (αGFP). Mice received 1 g/kg of glucose (i.p.) and blood glucose was measured at the indicated timepoints. Symbols correspond to individual mice and represent means ± SEM, *n* = 4-6 per experimental group; ***p < 0.001, ****p < 0.0001 from two-way ANOVA followed by Tukey’s *post-hoc* test comparing WT + αGFP vs. APP/PS1 + αGFP and APP/PS1 + αGFP vs. APP/PS1 + αIL6). **h** Plasma IL-6 in 11-month-old APP/PS1 mice after 4 i.c.v. injections of anti-IL-6 (αIL6) or anti-GFP (αGFP). Bars represent means ± SEM. Symbols correspond to individual mice, *n* = 8–12 per experimental condition; p-values were calculated from two-way ANOVA followed by Tukey’s *post-hoc* test and are shown in the graph. Data are representative of at least two independent experiments.

Next, we aimed to investigate the link between Aβ, hypothalamic IL-6 signaling and peripheral metabolic dysregulation in mouse models of AD. We found elevated IL-6 expression (Fig. 5a) and protein levels (Fig. 5b) in the hypothalamus of AβO-infused mice. Moreover, in situ hybridization revealed augmented SOCS3 mRNA density in the arcuate nucleus of the hypothalamus of mice infused with AβOs (Fig. 5c). A trend of increase in IL-6 (Fig. 5d) and increased SOCS3 protein levels (Fig. 5e) were further verified in the hypothalamus of APP/PS1 mice.

Finally, inhibition of brain STAT3 signaling by i.c.v. treatment with AG490 normalized peripheral glucose tolerance in AβO-infused mice (Fig. 5f), and i.c.v. treatment with anti-IL-6 reversed glucose intolerance in APP/PS1 mice (Fig. 5g). Moreover, while the central administration of anti-IL-6 did not alter plasma insulin or leptin (Supplementary Fig. 5a, b), this approach normalized the plasma concentrations of IL-6 in APP/PS1 mice (Fig. 5h).

## Discussion

Metabolic disorders are major risk factors for dementia^58–60^, and chronic systemic inflammation related to peripheral dysmetabolism may play a role in AD pathogenesis^10^. We observed T2-weighted MRI signal hyperintensities in the hippocampus and hypothalamus of AD patients compared to healthy control individuals, suggesting inflammation. It is plausible that the gliosis we observed in *post-mortem* AD hypothalamus may be linked to the inflammatory process revealed by imaging, as gliosis has been associated with increased T2-weighted signals in a number of studies^35,38,46,47^. Conversely, T2 hyperintensities can also emerge from other types of pathological substrates, such as white matter vascular lesions^61^, and independent investigations aiming to determine definitive proof of increased gliosis (i.e., positron-emission tomography and magnetic resonance spectroscopy) in the hypothalamus of living AD subjects are warranted.

While increased blood glucose is a risk factor for AD, our human sample did not present type 2 diabetes as a co-morbidity, indicating that the alterations observed in the hypothalamus are likely a consequence of AD pathogenesis. In addition, although previous volumetric analysis from imaging studies revealed hypothalamic atrophy in AD^48–50^, which is well known to occur in the cortex and hippocampus, the link between inflammation and brain structural changes remains to be better determined. Here, we provide initial evidence that gliosis may be associated with neurodegeneration. Additionally, resting-state functional MRIs (fMRI) demonstrated functional connectivity between the hippocampus and the hypothalamus^62^. Therefore, the strong T2 signal ratio correlation we observed between both brain regions in this study raises the possibility of an interconnected inflammatory process occurring in these two brain regions in AD.

Considering the heterogeneity of the hypothalamus, future detailed investigation of substructural pathology will be important to elucidate the contribution of specific hypothalamic nuclei to behavioral and metabolic alterations associated with AD. In our study, we detected amyloid plaques and neurofibrillary tangles (NFTs), key markers of AD pathology in the lateral hypothalamus of AD *post-mortem* tissue, which goes in line with early studies^63–65^. Hypocretin-1 loss was shown to occur in this same hypothalamic nucleus in AD^66^, which could be a culprit for sleep disturbances, commonly reported in the disease^67^. Disruptions of the sleep-awake cycle as a consequence of dysregulation of the lateral hypothalamus could play a major role in the cognitive decline and on behavioral symptoms that occur in AD^68^.

IL-6 has been negatively implicated in memory formation, as blocking IL-6 enhances long-term potentiation and improves long-term memory in a hippocampus-dependent task^69^. We found astrocytes surrounding both parenchymal and vascular amyloid deposits in AD brains and IL-6 immunoreactivity in astrocytes that were positive for Aβ. Further, we show that AD patients have elevated IL-6 in the brain and plasma, and that plasma IL-6 correlates positively with brain T2 hyperintensities and negatively with cognitive performance assessed by the MMSE and FAB tests. Wild-type mice exposed to exogenous oligomeric Aβ exhibited elevated IL-6, suggesting that Aβ is upstream to increased IL-6 production. Central administration of Aβ_1-42_ in mice has been shown to induce a systemic IL-6 response characterized by increased plasma and brain levels of this cytokine^70^. Here, we demonstrated that central acute blockade of IL-6 rescued glucose intolerance, plasma IL-6 concentration and memory performance in APP/PS1 mice, indicating a central role of IL-6 in memory and metabolic dysregulations in AD^71,72^. Collectively, these results suggest that the brain could be involved with the dysregulation of circulating IL-6 that occurs in AD, which could comprise the inflammatory reflex mediated by the vagus nerve^73^.

The central overexpression of IL-6 leads to an upregulation of TNF-α and microglial cells^74^, which are mediators of cognitive impairment, metabolic dysfunction and depressive-like behavior in AD mouse models^2,11,75^. However, the overexpression of IL-6 in the brains of TgCRND8 mice was shown to induce microglial activation and increase hippocampal Aβ clearance^76^. Therefore, the impact of chronic neutralization of IL-6 and its effects on neuroinflammation remains to be better determine. Follow-up studies aiming to longitudinally monitor IL-6 expression in association with astrogliosis and microgliosis will also help to elucidate the association between IL-6 and memory/metabolic impairments.

It has been well established by our group and by others that the accumulation of AβOs triggers tau hyperphosphorylation, NFTs^77^ and impaired insulin signaling. Impaired hippocampal insulin signaling is linked to memory deficits and cognitive decline in AD models and in patients^78–81^. In the hypothalamus, insulin resistance is linked to altered peripheral glucose homeostasis^2,82^. Tau loss of function as a consequence of hyperphosphorylation and NFTs formation was recently suggested to be involved in brain insulin resistance via insulin receptor substrate 1 (IRS-1) and phosphatase and tensin homolog (PTEN) alterations^83^. SOCS3 downregulates insulin signaling via ubiquitin-mediated degradation of the IRS-1^84^. In animal models of obesity, SOCS3 mediates insulin resistance in central and in peripheral tissues^85,86^. Therefore, given that SOCS3 is a downstream mediator of IL-6 signaling, studies aiming to determine whether the increased levels of this cytokine in AD elicits insulin signaling impairment via SOCS3 are warranted.

STAT3 is a transcription factor that regulates Aβ production and astrocyte proliferation and neurotoxicity^87^. Deletion of STAT3 in astrocytes decreases pro-inflammatory responses, improves Aβ clearance, and preserves memory in APP/PS1 mice^88^. We observed activation of brain STAT3 in AD mouse models, with strong nuclear staining in non-neuronal cells. In contrast, age-dependent reduction of pSTAT3 has been reported in the hippocampi of Tg2576 AD mice and AD subjects^57^. STAT3 promotes synaptic plasticity^89^ and neurogenesis^90^, processes centrally involved in learning and memory^91^. Thus, while the pharmacological inhibition of STAT3 by AG490 prevented memory decline in AβO-infused mice, it is noteworthy that AG490 per se caused memory impairment in control animals. The memory impairment caused by the pharmacological inhibition of STAT3 may be explained by an impairment of the leptin signaling^92^ and/or disruption of muscarinic acetylcholine receptor function and extracellular signal-regulated kinase (ERK) signaling^57^. Altogether, results indicate that memory function requires fine regulation of STAT3 phosphorylation levels.

Although the connection between peripheral and brain dysregulation in AD is not completely understood, alterations in BBB have been found in AD. Vascular Aβ deposition may impair the function of the BBB, which could increase the brain’s susceptibility to peripheral pro-inflammatory signals. Indeed, elevated Aβ is associated with impaired brain perfusion and BBB function^93,94^, features observed in metabolic disorders^7,95,96^. AD patients have high concentrations of circulating IL-6 and increases in brain IL-6 could result from local and/or systemic production.

Central and/or peripheral dysregulation of IL-6 is also associated with acute and chronic stress and with major depressive disorder^97^. Since the major depressive disorder is an important risk factor for developing AD later in life^98^, IL-6 emerges as an additional shared link between both diseases. Even though its contribution to AD pathogenesis remains to be fully elucidated, recognition of the potential role of brain inflammation in the onset and progression of AD has encouraged clinical trials targeting inflammation^99,100^. Our study supports the notion that inflammation is implicated in AD and suggests that elevated IL-6 production and signaling could be linked to cognitive decline and to peripheral metabolic dysfunction in AD.

## Supplementary information

Supplemental Material
