# Supplementary material for: Pro-inflammatory interleukin-6 signaling links cognitive impairments and peripheral metabolic alterations in Alzheimer’s disease
Source: Transl Psychiatry. 2021 Apr 28;11:251. doi: 10.1038/s41398-021-01349-z (PMC8080782; doi:10.1038/s41398-021-01349-z)
Supplement: Supplementary file 1 — Supplemental Material [file 41398_2021_1349_MOESM1_ESM.docx]

Supplementary Table 1: Demographics and characteristics of AD and control groups enrolled for MRI analysis.

|  | **Healthy controls** | **Alzheimer’s dementia** | **p test** |
| --- | --- | --- | --- |
|  | **(n=18)** | **(n=16)** |  |
| **Males n (%)** | 7 (38.9) | 8 (50.0) | 0.730^a^ |
| **Age (years)** | 66.3 [48.0, 81.0] | 70.2 [54.0, 84.0] | 0.101^b^ |
| **Education (years)** | 13.5 [10.2, 15.0] | 11.5 [8.0, 16.0] | 0.944^b^ |
| **Disease duration (years)** | - | 2.8 [1.0, 6.0] | - |
| **MMSE** | 29.1 [28.0, 30.0] | 24.1 [19.0, 28.0] | <0.0001^b^ |
| **Delayed recall score^c^** | 9.3 [8.0, 10.0] | 3.7 [0.0, 7.0] | <0.0001^b^ |
| **Category fluency/min** | 18.5 [13.0, 27.0] | 11.2 [3.0, 18.0] | <0.0001^b^ |
| **Verbal phonemic fluency** | 32.7 [16.0, 54.0] | 23.6 [4.0, 41.0] | 0.014^b^ |
| **Frontal Assessment Battery** | 15.5 [12.0, 18.0] | 12.7 [7.0, 17.0] | 0.001^b^ |
| **Hypertension n (%)** | 8 (50.0) | 1 (6.2) | 0.033^a^ |
| **Diabetes n (%)** | 3 (18.7) | 0 (0.0) | 0.269^a^ |
| **CSF Aβ42 (pg/mL)** | - | 592.4 [399.4, 723.7] | - |
| **CSF Tau (pg/mL)** | - | 684.2 [328.3, 1167.0] | - |
| **CSF pTau T181 (pg/mL)** | - | 92.3 [40.8, 150.6] | - |

Values represent means (percentile), means [range] and p-values from Fisher’s exact test (a) and Student’s t-test (b).

Supplementary Table 2: Demographics and characteristics of *post-mortem* tissue donors.

|  | **Controls** | **Alzheimer’s disease** |
| --- | --- | --- |
|  | **(n=9)** | **(n=8)** |
| **Males, n (%)** | 2 (22.2) | 6 (75.0) |
| **Age (years)** | 94.7 [72.0, 92.0] | 86.6 [78.0, 95.0] |
| **MMSE** | 28.1 [26.0, 30.0] | 17.75 [13.0, 24.0] |
| **PMI (hours)** | 2.9 [1.7, 4.3] | 3.3 [1.6, 5.0] |
| **BMI** | 26.0 [19.8, 33.7] | 24.7 [19.2, 30] |
| **Diabetes, n (%)** | 2 (22.2) | 1 (12.5) |
| **Depression, n (%)** | 1 (11.1) | 2 (25.0) |
| **Thyroid Disease, n (%)** | 4 (44.4) | 4 (50.0) |
| **Heart Failure, n (%)** | 3 (33.3) | 1 (12.5) |

Values represent means [range]. PMI, *post-mortem* interval; BMI, body mass index; MMSE, Mini Mental State Examination score.

Supplementary Table 3: List of primers used in qPCR analyses

| **Target gene** | **Forward primer** | **Reverse primer** |
| --- | --- | --- |
| *SOCS3* | GCGGGCACCTTTCTTATCC | TCCCCGACTGGGTCTTGAC |
| *IL6* | TTCTTGGGACTGATGCTGGTG | CAGAATTGCCATTGCACAACT |
| *β-actin* | GCCCTGAGGCTCTTTTCCAG | TGCCACAGGATTCCATACCC |
| *β-tubulin* | TAGACCCCAGCGGCAACTAT | GTTCCAGGTTCCAAGTCCACC |


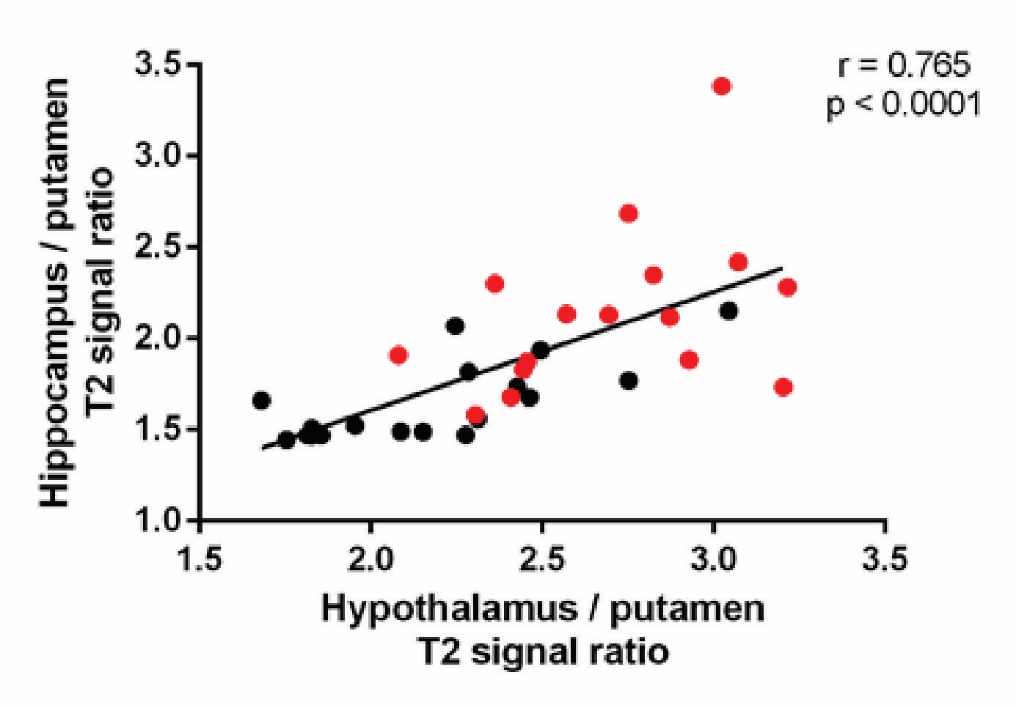


Supplementary Figure 1: Correlation between hippocampus/putamen and hypothalamus/putamen T2 hyperintensity signal ratios for patients (individual symbols) in our studied cohort (n=34). Spearman’s rank correlation coefficient (r) and p-value are shown.


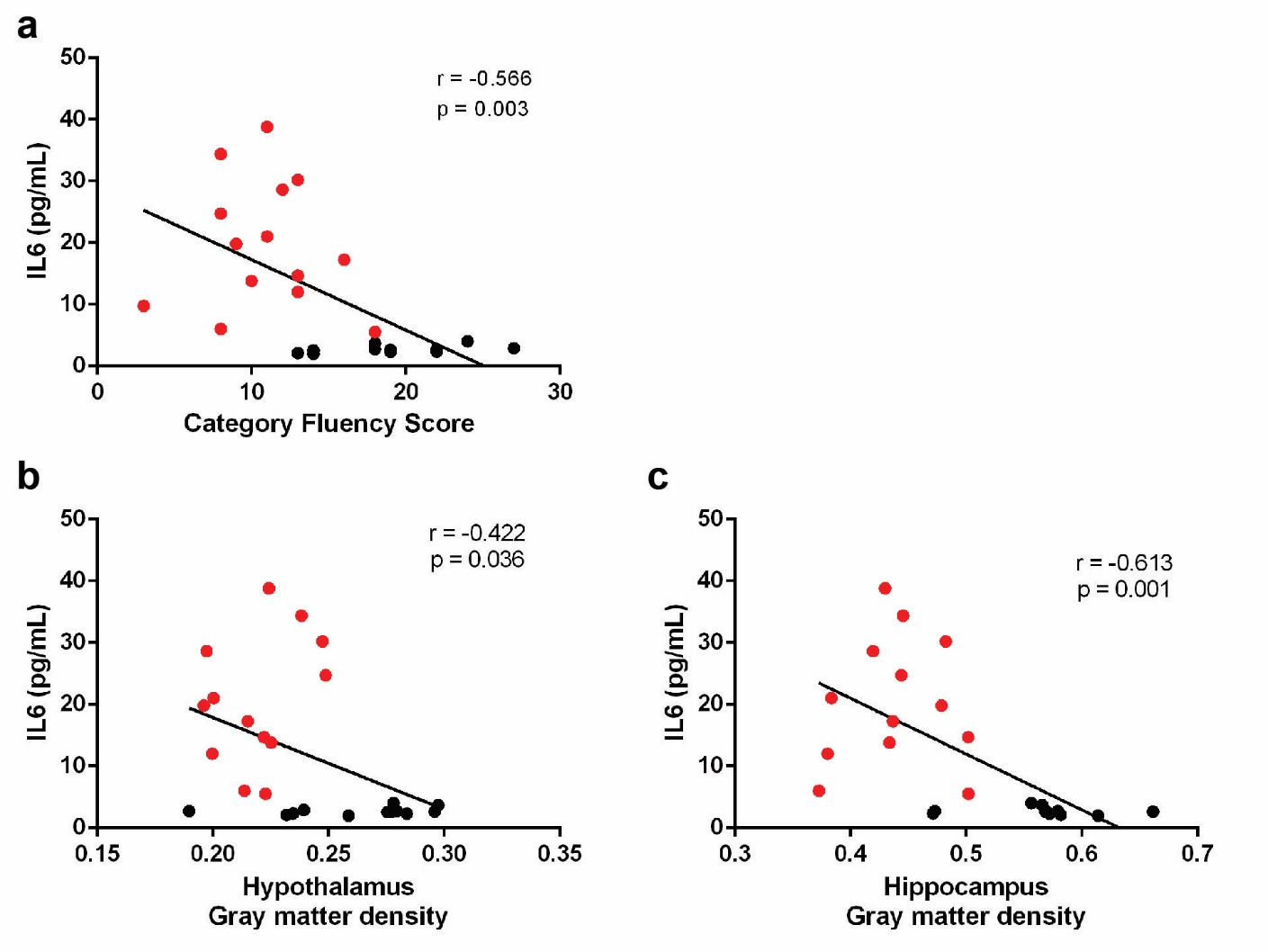


Supplementary Figure 2: Correlation between plasma IL-6 levels with Category Fluency test score (a) and hypothalamus (b) and hippocampus (c) gray matter densities for patients (individual symbols) in our studied cohort (n=25-26). Pearson correlation coefficient (r) and p-value are shown in the graphs.


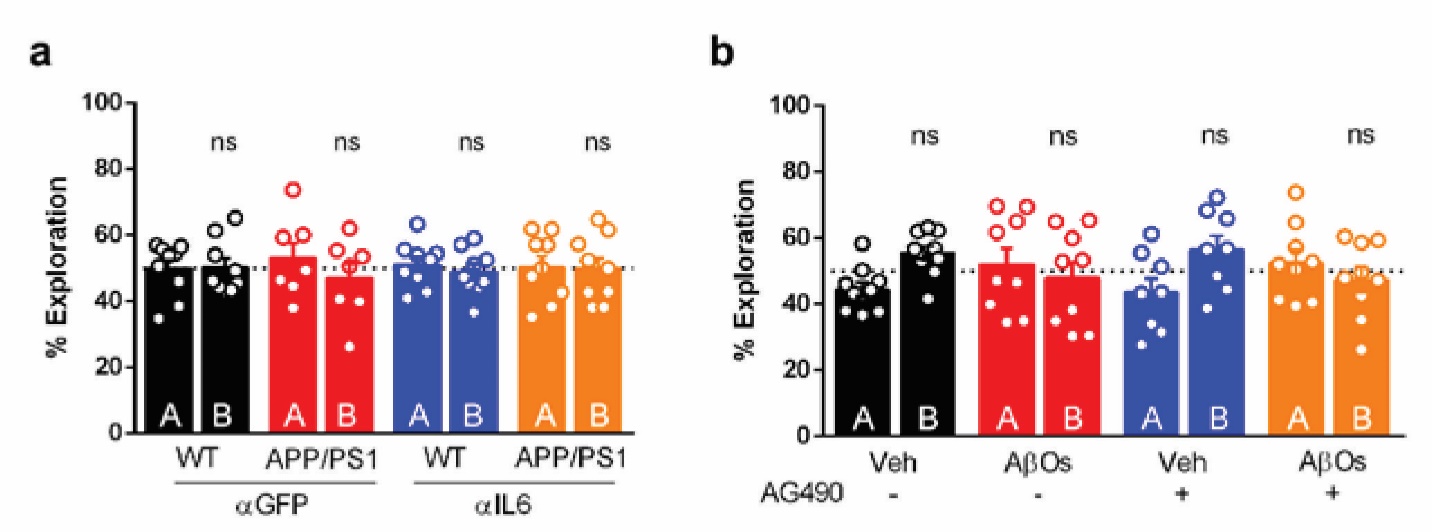


Supplementary Figure 3: Training phase of Novel Object Recognition test with 11-month-old male APP/PS1 mice and WT littermates after 3 i.c.v. injections of anti-IL-6 (αIL6) or anti-GFP (αGFP), as indicated (a) and with 3-month-old mice that received i.c.v. infusions of AβOs (or vehicle) and AG490, as indicated (b). Bars represent means +/- SEM and symbols represent individual values, n=7-10 per experimental condition. ns: not significant (one-sample Student’s t-test).


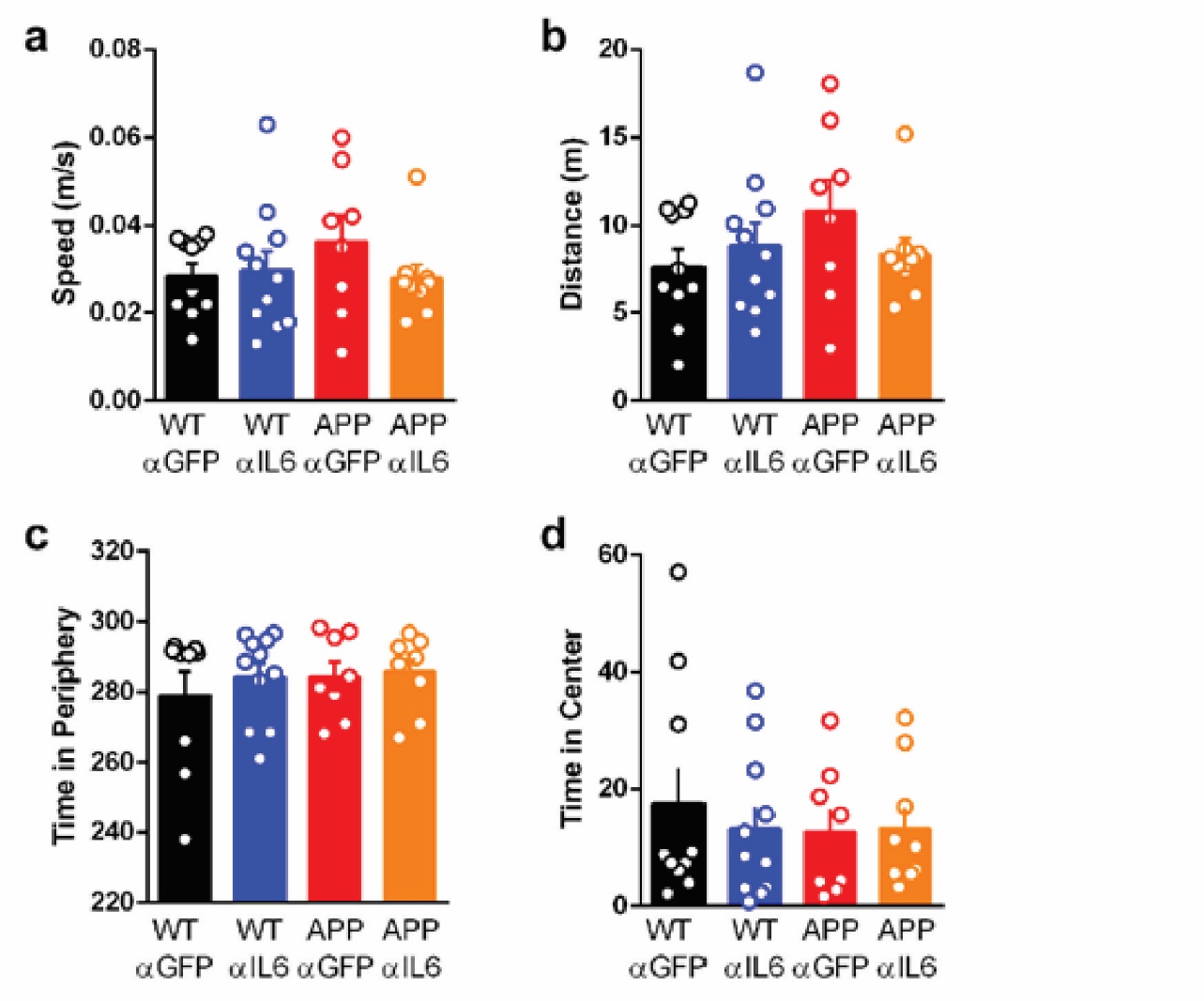


Supplementary Figure 4: Open Field test with 11-month-old male APP/PS1 mice and WT littermates after 3 i.c.v. injections of anti-IL-6 (αIL6) or anti-GFP (αGFP), as indicated. Graph shows mean +/- SEM and individual values of speed (a), distance (b), time in the periphery (c) and center (d) of experimental groups (n= 7-12 per experimental condition). ns: not significant (two-way ANOVA followed by Tukey`s *post-hoc* test).


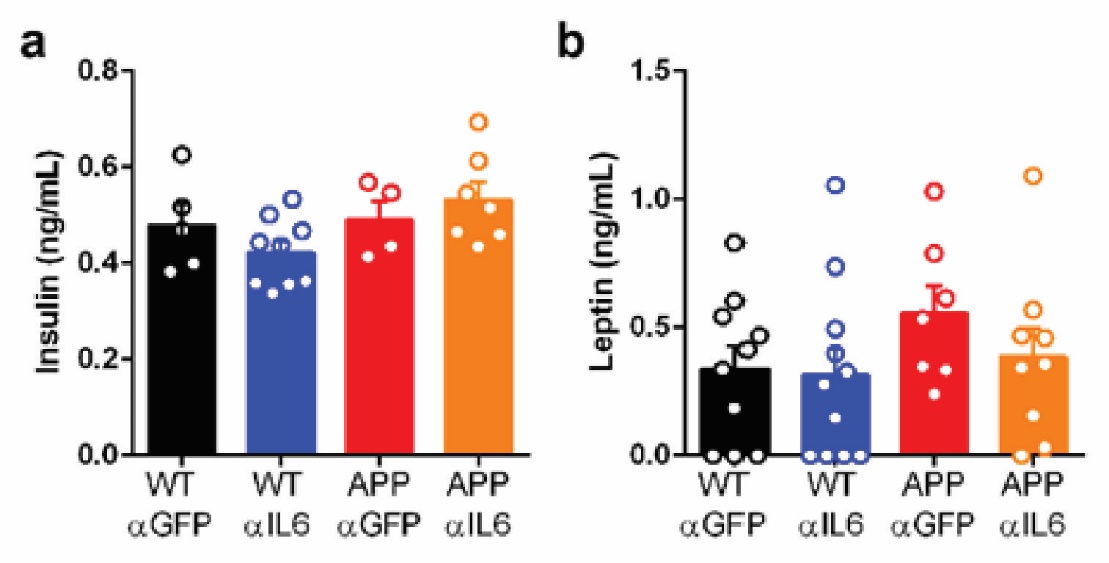


Supplementary Figure 5: Plasma levels of insulin (a) and leptin (b) in with 11-month-old male APP/PS1 mice and WT littermates after 3 i.c.v. injections of anti-IL-6 (αIL6) or anti-GFP (αGFP), measured by ELISA. Bars represent means +/- SEM and symbols represent individual values, n=4-11 per experimental condition. ns: not significant (two-way ANOVA followed by Tukey`s *post-hoc* test).
